# A new staging system for nasopharyngeal carcinoma based on intensity-modulated radiation therapy (IMRT)

**DOI:** 10.18632/oncotarget.21615

**Published:** 2017-10-07

**Authors:** Min Kang, Pingting Zhou, Jianxiong Long, Guisheng Li, Haolin Yan, Guosheng Feng, Meilian Liu, Jinxian Zhu, Rensheng Wang

**Affiliations:** ^1^ Department of Radiation Oncology, The First Affiliated Hospital of Guangxi Medical University, Radiation Oncology Clinical Medical Research Center of Guangxi, Nanning 530021, Guangxi, China; ^2^ School of Public Health, Guangxi Medical University, Nanning 530021, Guangxi, China; ^3^ Department of Radiation Oncology, Liuzhou Worker Hospital, Liuzhou 545000, Guangxi, China; ^4^ Department of Radiation Oncology, First People’s Hospital of Yulin City, Yulin 537000, Guangxi, China; ^5^ Department of Radiation Oncology, People’s Hospital of Guangxi Zhuang Autonomous Region, Nanning 530021, Guangxi, China; ^6^ Department of Radiation Oncology, Affiliated Hospital of Guilin Medical University, Guilin 541000, Guangxi, China; ^7^ Department of Radiation Oncology, Wuzhou Red Cross Hospital, Wuzhou 543000, Guangxi, China

**Keywords:** nasopharyngeal carcinoma (NPC), staging system, UICC/AJCC, magnetic resonance imaging (MRI), intensity-modulated radiation therapy (IMRT)

## Abstract

**Objective:**

This study is to establish a new staging system for nasopharyngeal carcinoma (NPC) based on the magnetic resonance imaging (MRI) and intensity-modulated radiation therapy (IMRT).

**Methods:**

Totally 492 patients with nasopharyngeal carcinoma were included in this study. These patients were diagnosed by pathological detection (without distant metastasis) and underwent the initial treatment of IMRT. These patients were subjected to the staging with the International Union against Cancer/American Joint Committee on Cancer (UICC/AJCC) staging system. Survival rates were calculated by the Kaplan-Meier method. Log-rank test was used to calculate the single factor prognosis, and the COX risk model was used to analyze the multivariate prognosis.

**Results:**

In these 492 patients, according to our recommended new T and N staging criteria, there were 290 cases of T1 and 202 cases of T2; there were 64 cases of N0, 159 cases of N1, 226 cases of N2, and 43 cases of N3. Univariate and multivariate analyses showed that the T and N staging combination parameters were independent prognostic factors, which affected the overall survival rates and tumor-free survival rates. According to risk difference and survival curve distribution, the following new clinical staging criteria were established: stage I (T1N0M0), stage II (T1N1M0 and T2N0M0), stage III (T1N2M0 and T2N1-2M0), stage IVa (T1-2N3M0), and stage IVb (TxNxM1).

**Conclusion:**

A new staging system for NPC based on MRI and IMRT has been recommended, which provides valuable evidence for disease treatment and prognosis prediction.

## INTRODUCTION

Over the past decade, the diagnosis and treatment of nasopharyngeal carcinoma (NPC) has undergone revolutionary changes. Magnetic resonance imaging (MRI), with high resolution for soft tissue than computed tomography (CT), could accurately define the extent of tumor invasion and allow for early detection of occult metastasis, which has been widely used in the clinical diagnosis of NPC [1–4]. For the disease treatment, compared with the conventional two-dimensional radiotherapy, the intensity-modulated radiation therapy (IMRT) could elevate the dosage in the target region while protect the normal tissues, which significantly improves the local control rate in the treatment of NPC in clinic and increases the 5-year survival rate to 80% [5].

In a large number of studies, the seventh edition of the International Union against Cancer/American Joint Committee on Cancer (UICC/AJCC) staging system based on conventional radiographic data has been shown to be deficient in predicting the prognosis of patients with NPC [6–8], and there are numerous studies concerning the staging systems of nasopharyngeal carcinoma [9–12]. A preliminary study from our laboratory has developed the new T and N staging systems for NPC according to the MRI and IMRT [13–15].

The new T staging system for NPC can be classified as T1 (nasopharynx, nasal cavity, parapharyngeal space, oropharynx, skull base and internal pterygoid muscle) and T2 (external pterygoid muscle, paranasal sinus, infratemporal fossa, orbit, cranial nerves, cavernous sinus and intracalvarium). The new N staging standards: N0 (no lymph node metastasis), N1 [retropharyngeal or/and unilateral upper cervical (I, II, III, Va, VIIb, VIII, IX, and X regions) lymph node metastasis], N2 [bilateral upper cervical (I, II, III, Va, VIIb, VIII, IX, and X regions) lymph node metastasis], and N3 (lymph node metastasis in IVa and Vb regions and their lower regions).

In this study, a new clinical staging system for nasopharyngeal carcinoma was proposed based on our proposed T and N staging system, to provide evidence for the disease diagnosis and prognosis prediction in clinic.

## RESULTS

### General survival of patients with nasopharyngeal carcinoma

For all these patients, the overall 5-year follow-up rate was 96.6%. Moreover, the 5-year OS, LRFS, DMFS, and DFS were 80.5%, 94.1%, 84.3%, and 78.6%, respectively. According to the UICC staging results (the 7^th^ edition), in all these 492 patients, the patients at the T1, T2, T3, and T4 stages accounted for 6.7% (33/492), 18.5% (91/492), 33.7% (166/492), and 41.1% (202/492), respectively; the percentages of patients at the N0, N1, N2, N3a, and N3b stages were 13.0%, 32.3%, 45.9%, 2.2%, and 6.5%, respectively; and the proportions of patients in the I, II, II, IVa, and IVb phases were 3.0% (15/492), 14.4% (71/492), 35.8% (176/492), 38.0% (187/492), and 8.7% (43/492), respectively. On the other hand, according to the new T and N staging criteria, the patients at the T1 and T2 stages accounted for 40.9% (201/492) and 59.1% (291/492), respectively; and the percentages of patients at the N0, N1, N2, and N3 stages were 13.0% (64/492), 32.3% (159/492), 45.9% (226/492), and 8.7% (43/492), respectively.

### Univariate and multivariate analyses of patients with nasopharyngeal carcinoma

In these 492 patients, univariate analysis was performed for the parameters including the gender, age, with or without chemotherapy, UICC staging (7^th^ edition), and recommended new T and N staging (i.e., T1N0M0, T1N1M0, T1N2M0, T1N3M0, T2N0M0, T2N1M0, T2N2M0, and T2N3M0). Meanwhile, the OS, LRFS, DMFS, and LRFS were used as observing indicators. Our results showed that, the OS and DFS were significantly affected by the T1N0M0, T1N1M0, T1N2M0, T1N3M0, T2N0M0, T2N1M0, T2N2M0, and T2N3M0 (Table 1) (*P* < 0.05), which were therefore involved in the following multivariate analysis.

**Table 1:** Univariate analysis of disease prognosis of nasopharyngeal carcinoma

	N	Overall survival (OS) rate	*P*	Local relapse -free survival (LRFS) rate	*P*	Distant metastasis-free survival (DMFS) rate	*P*	Disease-free survival (DFS) rate	*P*
Gender									
Male	338	79.0%	0.750	94.9%	0.294	84.2%	0.713	78.4%	0.683
Female	154	80.5%		97.1%		83.7%		80.5%	
Age									
≥ 45 years	272	82.7%	0.094	93.1%	0.549	89.9%	0.000	83.8%	0.011
< 45 years	220	75.3%		95.4%		76.6%		73.0%	
Treatment									
IMRT	31	93.5%	0.048	96.8%	0.512	96.8%	0.050	93.5%	0.043
IMRT + chemicaltherapy	461	78.5%		94.0%		83.2%		78.1%	
UICC staging									
Stage I	15	93.3%	0.000	93.3%	0.004	93.3%	0.000	93.3%	0.000
Stage II	71	98.6%		93.0%		94.3%		93.0%	
Stage III	176	81.8%		98.1%		83.8%		83.0%	
Stage IVa	187	75.9%		87.9%		83.1%		73.3%	
Stage IVb	43	48.8%		94.7%		53.5%		48.8%	
Novel T and N staging									
T1N0M0	29	97.6%	0.045	97.6%	0.063	100%	0.492	97.6%	0.049
T1N1M0	110	90.5%	0.041	98.1%	0.014	95.6%	0.000	89.7%	0.021
T1N2M0	49	77.7%	0.018	98.8%	0.020	77.5%	0.107	76.6%	0.038
T1N3M0	13	54.5%	0.001	70.0%	0.052	57.6%	0.001	54.5%	0.001
T2N0M0	35	89.5%	0.027	94.4%	0.764	89.5%	0.438	89.5%	0.028
T2N1M0	49	77.3%	0.007	90.5%	0.531	84.1%	0.085	70.5%	0.018
T2N2M0	177	74.8%	0.003	86.7%	0.000	78.9%	0.605	73.3%	0.007
T2N3M0	30	42.9%	0.001	79.1%	0.057	50.0%	0.001	42.9%	0.001

For the multivariate analysis, the OS and DFS were used as observing indicators, and meanwhile the gender, age, and with or without chemotherapy were applied as covariants for correction. As shown in Table 2, our results showed that, compared with patients at the T1N0M0 (HR=1) stage, there were significant differences in the relative death risk and tumor-free ratio for the patients at the T1N1-3M0 and T2N0-3M0 stages (*P* > 0.05). Accordingly, the T1N0M0 stages could be considered as stage I. compared with patients at the T1N1M0 (HR=1) stage, there were no significant differences in the relative death risk and tumor-free ratio for the patients at the T2N0M0 stages (*P* > 0.05). Accordingly, the T1N1M0, and T2N0M0 stages could be together considered as stage II. Compared with patients in the stage II (HR=1), there were significant differences in the death risk and tumor-free ratio for the patients at the stages of T1N2M0, T1N3M0, T2N1M0, T2N2M0, and T2N3M0 (*P* < 0.05). Moreover, if the T1N2M0 stage was considered as baseline (HR=1), there were no significant differences in the death risk and tumor-free ratio for the patients at the stages of T2N1M0 and T2N2M0 (*P* > 0.05). Accordingly, the stages of T1N2M0, T2N1M0, and T2N2M0 could be combined into the stage III. Compared with the patients in the stage III, there were significant differences in the death risk and tumor-free ratio for the patients at the T1N3M0 and T2N3M0 stages (*P* < 0.05). Furthermore, there were no significant differences in the death risk and tumor-free ratio between the patients at the T1N3M0 and T2N3M0 stages, and therefore the T1N3M0 and T2N3M0 could be considered as the stage IVa. Taken together, a new clinical staging system has been established: stage I (T1N0M0), stage II (T1N1M0 and T2N0M0), stage III (T1N2M0 and T2N1-2M0), stage IVa (T1-2N3M0), and stage IVb (TxNxM1).

**Table 2:** Death risks and tumor-free rates based on recommended T and N staging system

		5y-OS		5y-DFS	
		P	HR(95%CI)	P	HR(95%CI)
T1N0M0 VS	T1N1M0	0.013	2.313 (1.192-4.489)	0.019	2.197 (1.139-4.236)
	T1N2M0	0.017	5.887 (1.380-25.108)	0.013	6.320 (1.486-26.878)
	T1N3M0	0.001	13.144 (2.986-57.855)	0.000	14.367 (3.284-62.853)
	T2N0M0	0.001	3.828 (1.694-8.652)	0.002	3.533 (1.572-7.943)
	T2N1M0	0.021	6.000 (1.315-27.383)	0.006	7.930 (1.789-35.146)
	T2N2M0	0.008	6.810 (1.634-28.382)	0.006	7.350 (1.768-30.562)
	T2N3M0	0.000	24.477 (5.284-113.386)	0.000	21.579 (4.660-99.925)
T1N1M0vs	T1N2M0	0.010	2.615 (1.261-5.423)	0.009	2.570 (1.272-5.194)
	T1N3M0	0.000	6.151 (2.823-13.402)	0.000	5.751 (2.690-12.296)
	T2N0M0	0.867	1.138 (0.252-5.132)	0.953	1.046 (.234-4.672)
	T2N1M0	0.025	2.665 (1.132-6.275)	0.003	3.222 (1.470-7.062)
	T2N2M0	0.001	3.025 (1.528-5.985)	0.001	2.988 (1.551-5.758)
	T2N3M0	0.000	10.124 (4.067-25.199)	0.000	9.270 (3.784-22.706)
T2N0M0+T1N1M0 VS	T1N2M0	0.008	2.566(1.285-5.125)	0.006	2.554(1.307-4.992)
	T1N3M0	0.000	6.036(2.870-12.696)	0.000	5.715(2.756-11.850)
	T2N1M0	0.022	2.615(1.147-5.964)	0.003	3.202(1.505-6.812)
	T2N2M0	0.001	2.968(1.562-5.640)	0.001	2.970(1.598-5.520)
	T2N3M0	0.000	9.936(4.113-24.000)	0.000	9.212(3.860-21.987)
T1N2M0 VS	T1N3M0	0.022	2.199 (1.118-4.326)	0.016	2.248 (1.166-4.335)
	T2N1M0	0.956	1.021 (0.481-2.169)	0.527	1.248 (0.628-2.477)
	T2N2M0	0.603	1.156 (0.669-1.998)	0.585	1.160 (0.681-1.978)
	T2N3M0	0.000	4.080 (1.866-8.920)	0.002	3.379 (1.555-7.341)
T1N2M0+T2N1M0+T2N2M0 VS	T1N3M0	0.016	2.038 (1.143-3.636)	0.014	2.010 (1.150-3.511)
	T2N3M0	0.000	3.782 (1.879-7.610)	0.002	3.021 (1.508-6.050)

### Survival curves based on recommended new clinical staging combination parameters

**Figure 1:**
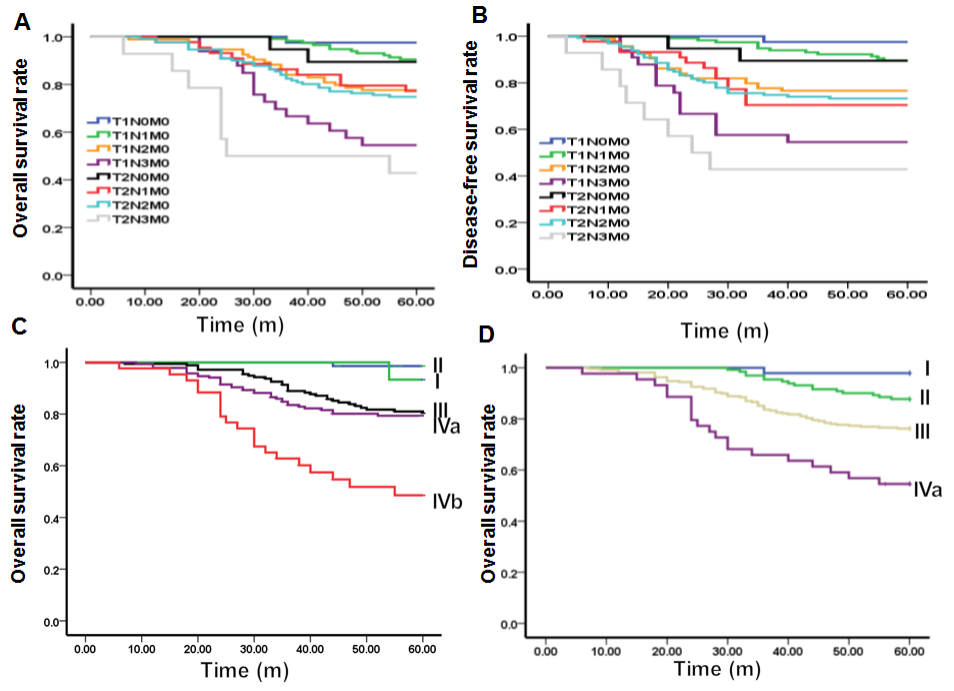
Survival curves based on the recommended new clinical staging system and the UICC/AJCC staging system (the 7^th^ edition) The overall survival curves **(A)** and tumor-free survival curves **(B)** based on the recommended new T and N staging system combination parameters. The overall survival curves based on the UICC/AJCC staging system (the 7^th^ edition) **(C)** and the overall survival curves based on the recommended new clinical staging system **(D)**.

The overall survival curves and tumor-free survival curves were constructed based on the recommended new clinical staging combination parameters, including T1N0M0, T1N1M0, T1N2M0, T1N3M0, T2N0M0, T2N1M0, T2N2M0, and T2N3M0. As shown in Figure 1, our analysis showed that, comparable overall survival curves and tumor-free survival curves were observed for T1N0M0, there were statistically significant differences with T1N1-3M0 and T2N0-3M0 stages (P<0.005), while, as observed for T1N1M0, and T2N0M0, with no statistically significant differences in overall survival curves and tumor-free survival curves (*P* > 0.05). Moreover, similar overall survival curves and tumor-free survival curves were observed for T1N2M0, T2N1M0, and T2N2M0, with no statistically significant differences (*P* > 0.05). Furthermore, comparable overall survival curves and tumor-free survival curves were observed for T1N3M0 and T2N3M0, with no statistically significant differences (*P* > 0.05). These results were in line with the results from the above univariate and multivariate analyses.

### Establishment and evaluation of new clinical staging system for nasopharyngeal cancer

According to the results from the univariate and multivariate analyses, as well as the survival curve analysis, the following new clinical staging criteria for the nasopharyngeal cancer based on the MRI and IMRT were established: stage I, T1N0M0; stage II, T1N1M0 and T2N0M0; stage III, T1N2M0 and T2N1-2M0; stage IVa, T1-2N3M0; and stage IVb, TxNxM1.

Next, the evaluation of the newly established staging system was performed (Table 3). For the survival prediction, as shown in Figure 1, there were significant differences in the overall survival curve between stages I and II (x^2^ = 4.054, *P* =0.044), stages II and III (x^2^ = 8.013, *P* = 0.005), as well as between stages III and IVa (x^2^ = 10.820, *P* = 0.001). However, in the UICC/AJCC staging system (7^th^ edition), no significant differences could be distinguished in the overall survival between stages I and II (*P* = 0.235), as well as between stages III and IVa (*P* = 0.431).

**Table 3:** Comparison of distribution balance and risk difference between the recommended novel staging and the UICC staging (the 7^th^ edition) systems

	N	Overall survival hazard ratio (95%CI)
Novel T and N staging		
Phase I	29 (5.8%)	1
Phase II	145 (29.4%)	2.009 (1.595-2.531)^*^
Phase III	275 (55.8%)	3.362 (1.885-5.994)^*^
Phase IVa	43 (8.7%)	8.495 (4.367-16.525)^*^
UICC staging		
Stage I	15 (2.9%)	1
Stage II	71 (13.5%)	1.311 (0.358-4.810)
Stage III	176 (33.5%)	3.165 (0.432-23.171)
Stage IVa	187 (35.6%)	3.824 (0.524-27.883)
Stage IVb	43 (8.7%)	11.996 (1.611-89.351)^*^

Note: ^*^, *P*< 0.05.

Moreover, for the distribution equilibrium, in our new staging system, the patients in the stages I, II, III and IVa were 29(5.8%), 145 (29.4%), 275 (55.8%), and 43 (8.7%), respectively. However, in the UICC/AJCC staging system (7^th^ edition), the patients in stage I only accounted for 2.9%, while the percentage of patients in stage II was 13.5%. Most patients were accumulated in stages III (33.5%) and IVa (35.6%).

Furthermore, for the risk difference analysis, when stage I was considered as baseline (HR = 1), in the new staging system, the total death risk was increased along with the stage development, with significant differences compared with the baseline (*P* < 0.05). However, in the UICC/AJCC staging system (7^th^ edition), no significant difference was observed in the total death risk for patients in stages II and III as compared with patients in stage I. Taken together, these results suggest that, in the terms of survival prediction, distribution equilibrium, and risk difference analysis, our new staging system was superior to the UICC/AJCC staging system (7^th^ edition).

## DISCUSSION

Basically, an ideal staging system should meet the following standards: (1) the survival rates for the patients within the same group should be similar; (2) the survival rates for the patients from different groups should be significantly different; (3) the proportions of patients in each group should be relatively balanced; and (4) the prognosis prediction should be accurate. The purpose and significance of clinical staging include the followings: (1) to guide the development of treatment programs; (2) to predict the prognosis; (3) to help to assess the treatment efficacy; (4) to facilitate the exchange and comparison of treatment data; and (5) to contribute to the investigation of human cancers and other diseases [16]. Accordingly, the TNM clinical staging system of nasopharyngeal carcinoma should have kept changing along with the development of diagnostic and treatment technologies. However, the 7^th^ edition of the UICC/AJCC staging standard (published in 2009) is primarily based on data from conventional two-dimensional radiotherapy, which does not reflect the impact of diagnosis and treatment technology development on disease staging [4, 17, 18]. Currently, there is still no clinical staging system concerning the radiation therapy. Therefore, it is particularly important to establish a new clinical staging system for nasopharyngeal carcinoma based on the IMRT.

Previous studies have showed that the invasions in the nasal cavity, pharynx oralis, parapharyngeal space, medial pterygoid, lateral pterygoid, infratemporal fossa, paranasal sinuses, orbit, intracalvarium, and cranial nerves are all in-dependent prognostic factors [13, 14]. According to the risk difference and survival curve distribution analyses, it is suggested that the new clinical T staging standards for nasopharyngeal carcinoma based on MRI and IMRT should include: T1, nasopharynx, pharynx, oropharynx, nasal cavity, skull base, and medial pterygoid; and T2, lateral pterygoid, cavernous sinus, paranasal sinuses, infratemporal fossa, orbit, intracalvarium, and cranial nerves. Based on these standards, the OS and LRFS for these nasopharyngeal carcinoma patients could be appropriately distinguished. Levels and numbers of retropharyngeal and cervical lymph nodes represent independent prognostic factors affecting the survival rates of nasopharyngeal carcinoma. According to the distant metastasis risks of the combinations and the risk congruence principle, the new N staging standards for the nasopharyngeal carcinoma: N0, with no lymph node metastasis; N1, lymph node metastasis in the VIIa region and/or unilateral upper cervical region (I, II, III, and Va regions); N2, lymph node metastasis in the bilateral upper cervical regions (I, II, III, and Va regions); and N3, lymph node metastasis in the IVa and Vb regions, as well as the lower regions. The OS and LRFS for these nasopharyngeal carcinoma patients could also be distinguished according to these N staging standards. Moreover, it is more simple and reasonable to apply the 2013 RTOG cervical partitioning criteria for the N staging. Based on the background, totally 492 patients with nasopharyngeal carcinoma undergoing IMRT from six treatment centers were included in this study. Considering the previously recommended T and N staging systems, the relationship between these parameter combinations and the patient prognosis was investigated, and a new clinical staging system for nasopharyngeal carcinoma based on MRI and IMRT data was established.

Our results showed that there were significant effects of parameter combinations from the recommended new T and N staging system on the overall survival rate and tumor-free survival rate of these patients with nasopharyngeal carcinoma. There were similar OS and DFS curves between T1N1M0, and T2N0M0. Moreover, compared with T1N0M0, the death risks and tumor-free rates were significant different for T1N1M0 and T2N0M0. Therefore, the T2N0M0 and T1N1M0 stages were combined into stage II. As the IMRT increases the dosage in the tumor target region, and improves the local control rate of nasopharyngeal carcinoma, indicating that the development of diagnosis and treatment technologies can reduce the impact of T staging on the disease prognosis and makes differences smaller between the patients from different T staging-based subgroups. These results are in line with the findings from Tham *et al.* [19] and Mao *et al.* [20]. Moreover, Zhao *et al.* [21] have reported the follow-up outcomes of 419 cases of IMRT, and the 5-year local control rate and regional local control rate are 92.7% and 95.8%, respectively. The multivariate analysis showed that the T staging did not represent the prognostic factor affecting the survival rate. Meanwhile, compared with the patients in the new stage II, the death risks and tumor-free rates were significantly higher for the T1N2M0 and T2N1-2M0 stages. Considering that patients at the T1N2M0 and T2N1-2M0 stages shared similar death risks and tumor-free rates, these stages were combined into stage III. In a report from Li *et al.* [22], the international cervical lymph node imaging region has been divided into the upper and lower cervical regions, with the lower edge of annular cartilage as the dividing line, and they have found that the metastatic risk is significantly increased for the distant area in the lower cervical region. In line these findings, our results showed that, compared with the patients in the stages I, II and II, the death risks were dramatically higher for the patients at the T1N3M0 and T2N3M0 stages. Accordingly, the stages of T1-2N3M0 were combined into the stage IVa. In our new staging system, the 5-year overall survival rate for patients in the stage IVa was 51.%. These results suggest that the nasopharyngeal carcinoma patients with lower cervical lymph node metastasis might be associated with poor disease prognosis.

In order to more objectively evaluate the prediction of prognosis of patients receiving IMRT through the T and N staging in the UICC (the 7^th^ edition), the patients with initial metastasis were not included in this study. Therefore, the survival status of the patients with metastasis could not be accurately assessed. Previous studies have shown that the survival time of the nasopharyngeal carcinoma patients with distant metastasis is relatively short, and the 5-year survival rate is less than 20%; the median survival time for the untreated patients is about 6-8 months, and the median survival time for those who received chemotherapy is only 8-12 months [23–25]. Based on these finding, in our new staging system, the TxNxM1 stages might be recommended to be combined into the stage IVb. For the patients in stage IVb, comprehensive evaluation (also considering the patients’ conditions) should be performed during the disease treatment.

The recommended new staging system was based on the MRI and IMRT background, and the overall survival rates for the patients in stages I, II, III and IVa were 97.6%, 87.8%, 76.2%, and 51.1%, respectively, with statistically significant differences in the overall survival curves between these patients, which matched with the staging principle. However, due to the limited sample size and the fact that nasopharyngeal carcinoma patients with initial metastasis were not included in this study, further in-depth studies are still needed in the future.

In conclusion, our results showed that the T and N staging combination parameters were independent prognostic factors affecting the overall survival rates and tumor-free survival rates. According to risk difference and survival curve distribution, the following new clinical staging criteria were established: stage I (T1N0M0), stage II (T1N1M0 and T2N0M0), stage III (T1N2M0 and T2N1-2M0), stage IVa (T1-2N3M0), and stage IVb (TxNxM1). The recommended new staging system for nasopharyngeal carcinoma is based on MRI and IMRT, which could provide valuable evidence for objective prognosis prediction and disease treatment.

## MATERIALS AND METHODS

### Study subjects

Totally 492 patients with nasopharyngeal carcinoma, who were admitted to six treatment centers in the Guangxi Zhuang Autonomous Region from January 2006 to December 2009, were included in this study. These patients were diagnosed by pathological detection (without distant metastasis) and underwent the initial treatment of IMRT. There were 338 males and 154 females, with the male-to-female ratio of 2.2 : 1, and the age range was 18-81 years old, with the median age of 45 years old. All these patients were subjected to detailed physical examination, general condition evaluation, hemogram analysis, nasopharyngeal fiberscopy, chest radiograph, abdominal B ultrasound, and MRI for nasopharyngeal + cervical soft tissue. The patients at the N2-N3 stages received the full-body bone scan. Prior written and informed consent were obtained from every patient and the study was approved by the ethics review board of Guangxi medical university.

### MRI scanning

MRI scanning was performed using the Signa 1.5t mr/i superconducting MRI instrument (GE, Milwaukee, WI, USA). Routine and enhanced scanning was conducted for all the cases. Scanning directions included the cross, sagittal, and coronal planes, involving T2WI (TR, 3000-4000 ms and TE, 102-110 ms), T1WI (TR, 2200-2400 ms; TE, 77-109 ms; and TI, 750 ms), and T1WI enhanced scanning (with the same scanning position and parameters with T1WI). Skull orthogonal coil was used, with the layer thickness of 6 mm, layer spacing of 1 mm, and the 256×192 matrix. Cross-sectional scanning was from the suprasellar cistern to the lower edge of clavicle. The contrast agent was gadopentetate dimeglumine (Gd-DTPA), with a total dose of 15-20 ml.

### Recommended new T and N staging criteria

The new T staging criteria included [13, 14]: T1, invasion in nasopharynx, parapharyngeal space, oropharynx, nasal cavity, skull base, and medial pterygoid; and T2, invasion in lateral pterygoid, paranasal sinuses, orbit, intracalvarium, infratemporal fossa, and cranial nerves. On the other hand, the new N staging criteria were as follows [15]: N0, with no lymph node metastasis; N1, lymph node metastasis in the VIIa region and/or unilateral upper cervical region (I, II, III, and Va regions); N2, lymph node metastasis in the bilateral upper cervical regions (I, II, III, and Va regions); and N3, lymph node metastasis in the IVa and Vb regions, as well as the lower regions.

### T and N staging system

According to the UICC/AJCC staging system for nasopharyngeal carcinoma (the 7^th^ edition), the nasopharyngeal and cervical MRI images in the PACS system were reviewed for all the patients. Combined with the symptoms and signs as admission (such as hemp, diplopia, and tongue flexion), the patients were re-staged. Lymph node metastasis was confirmed based on the MRI results. The locations of lymph nodes were determined based on the internationally accepted RTOG partition in 2013.

### Therapeutic method

Totally 492 NPC patients received IMRT during the whole process. Computed tomography contrast-enhanced scanning was applied from the skull cap to 3 cm below clavicle, with a layer distance of 3 mm and layer thickness of 3 mm. Under the guidance of Report 50 and Report 62 of International Commission on Radiation Units and Measurements (ICRU), gross tumor volume (GTV) included primary tumor sites and their invasion range (GTVnx), retropharyngeal metastatic lymph nodes (GTVrpn), and cervical metastatic lymph node (GTVnd). Clinical target volume (CTV) range can be adjusted according to involvement degrees. For example, CTV1 should include GTVnx, GTVrpn, the whole nasopharyngeal mucosa, and submucosal region (5 mm); CTV2 should include CTV1, as well as some of the following: posterior nasal cavity, pterygopalatine fossa, posterior maxillary sinus, part of the posterior ethmoid sinus, lateral pharyngeal space, skull base, part of cervical vertebra, and slope. Planning target volume (PTV) included position errors and organ movements during treatments, which are usually externally expanded for 3-5 mm based on GTVs and CTVs. The prescription doses were as follows: PGTVnx and PTVrpn (68-74 Gy), PTVnd (66-70 Gy), PTV1 (60-66 Gy), and PTV2 (50-56 Gy) (5 times/week for a total of 30-33 times). The setting of restricted dosages for critical organs followed international consensus [26, 27].

All stages were defined according to the 7th edition of the UICC/AJCC staging standards. Of the 477 patients with Stage II-IVB disease, 93.70% patients (461/492) received chemotherapy, including 51.0% (235/461) with concurrent chemotherapy, 37.09% (171/461) with induction + concurrent chemotherapy, 7.59% (35/461) with concurrent + adjuvant chemotherapy, 4.12% (19/461) with induction + concurrent + adjuvant chemotherapy, and 0.22% (1/461) with induction chemotherapy. The chemotherapy drugs were mainly platinum-based. All centers used identical chemotherapy protocols.

### Patient follow-up

Random follow-up started from 3 months after the treatment was completed. The follow-up period was defined as the period starting from the commencing date of treatment to the last date of follow-up, or to the patient death. Follow-up ended on May 31, 2016. Follow-up period was 6-77 months, with the median period of 58 months and the follow-up rate of 96.6%. Main analysis indicators included the overall survival (OS), disease-free survival (DFS), Local relapse-free survival (LRFS), and distant metastasis -free survival (DMFS).

### Statistical analysis

SPSS 19.0 software was used for comparison. Kaplan-Meier method was used to calculate the survival rate. Log-rank test was used to calculate the single factor prognosis, and the COX risk model was used to analyze the multivariate prognosis. *P* < 0.05 was considered as statistically significant.

## Abbreviations

NPC: nasopharyngeal carcinoma
MRI: magnetic resonance imaging
IMRT: intensity-modulated radiation therapy
ICRU: International Commission on Radiation Units and Measurements
GTV: gross tumor volume
CTV: clinical target volume
PTV: planning target volume
OS: overall survival
DFS: disease-free survival
RFS: relapse-free survival
DMFS: distant metastasis -free survival
LRFS: local relapse-free survival
